# Unclassifiable Isolated Monoclonal Lymphocytosis: Comprehensive Description of a Retrospective Cohort

**DOI:** 10.3390/cancers11101495

**Published:** 2019-10-04

**Authors:** Michaël Degaud, Lucile Baseggio, Béatrice Grange, Delphine Manzoni, Sarah Huet, Evelyne Callet-Bauchu, Alexandra Traverse-Glehen, Frédéric Davi, Hervé Ghesquières, Gilles Salles, Pierre Sujobert

**Affiliations:** 1 Hospices Civils de Lyon, Centre Hospitalier Lyon Sud, Laboratoire d’hématologie, 69495 Pierre Bénite, France; 2 INSERM 1052, CNRS 5286, Université Claude Bernard, Faculté de Médecine Lyon-Sud Charles Mérieux, Université de Lyon, 69495 Pierre Bénite, France; 3 Hospices Civils de Lyon, Centre Hospitalier Lyon Sud, Laboratoire d’anatomopathologie, 69495 Pierre Bénite, France; 4 Assistance Publique—Hôpitaux de Paris, Hôpital Pitié-Salpêtrière, Département d’hématologie biologique, Sorbonne Université, 75013 Paris, France; 5 Hospices Civils de Lyon, Centre Hospitalier Lyon Sud, Service d’hématologie clinique, 69495 Pierre Bénite, France

**Keywords:** WHO classification, lymphocytosis, B-cell neoplasms, chronic lymphocytic leukemia, marginal zone lymphoma

## Abstract

According to the World Health Organization (WHO) classification, the nosology of B-cell neoplasms integrates clinical, morphological, phenotypic, and genetic data. In this retrospective analysis, we identified 18 patients with isolated neoplastic lymphocytosis that could not be accurately classified within the WHO classification. Most of them were asymptomatic at the time of diagnosis and the evolution was relatively indolent, as only five patients required treatment after a median follow-up of 48 months. The neoplastic B-cells expressed CD5 in most cases, but the Royal Marsden Hospital score was strictly below 3. Trisomy 12 was the most frequent cytogenetic abnormality. High-throughput sequencing highlighted mutations found in both chronic lymphocytic leukemia (CLL) and marginal zone lymphoma (MZL). Similarly, the immunoglobulin heavy chain variable region repertoire was distinct from those reported in CLL or MZL. However, as treatment choice is dependent on the correct classification of the lymphoproliferative disorder, a histological diagnosis should be performed in case patients need to be treated.

## 1. Introduction

As stated in the fourth edition of the World Health Organization (WHO) classification of hematological malignancies, “classification is the language of medicine” [1]. The classification of lymphoid neoplasms integrates clinical, morphological, phenotypic, and genetic data to associate a malignant cell to its supposed normal counterpart. An ideal classification system should minimize overlap between categories, and every patient should be assigned to one (and only one) category after extensive characterization of the disease [1]. Given the huge variability in the outcome of lymphoid neoplasms and their clinical management, the precise refinement of their classification is ongoing work that is of utmost importance. The accuracy of classification is even more important with the use of targeted therapies, as their authorization is restricted to specific nosological entities with a given molecular alteration [2].

Lymphocytosis (defined as an absolute lymphocyte count above 5 G/L) is a frequent clinical presentation of B-cell neoplasms. Lymphocytosis can represent the dissemination in the blood of an overt lymphoma, or can be the only detectable manifestation of the neoplasm. In the latter case, accurate diagnosis relies exclusively on the characterization of the circulating lymphocytes, based on cytology, immunophenotyping, cytogenetics, and/or molecular biology. For instance, the diagnosis of chronic lymphocytic leukemia (CLL) requires a Royal Marsden Hospital (RMH) score [3,4] of three or more. The diagnosis of mantle cell lymphoma (MCL) relies on the demonstration of the t(11;14)(q13;q32) translocation leading to cyclin D1 overexpression [1]. Some patients present an isolated monoclonal lymphocytosis that does not fulfill the diagnostic criteria for any lymphoid neoplasm and thus cannot be accurately classified within the WHO classification. In order to describe the clinical and biological characteristics of these patients, we performed a retrospective single-center study of 18 patients with unclassifiable isolated absolute lymphocytosis.

## 2. Results

### 2.1. Clinical and Biological Characterization of the Cohort

The study included 18 cases (10 women and 8 men; Table 1), consulting for absolute lymphocytosis above 5 G/L without clinically detectable enlarged lymph node, splenomegaly, or hepatomegaly. This was confirmed by imaging (i.e., ultrasound, radiography, and/or scanner) in 12 patients. The median age at diagnosis was 79 years (range: 55–92 years). With the exception of one patient who had a moderately altered performance status (Eastern Cooperative Oncology Group (ECOG) score: 1), all patients were asymptomatic. The median lymphocytosis was 9.55 G/L (range: 5.30–25.69 G/L).

The cytological analysis of blood smears was performed for 17 patients, and the three types of lymphocytes (CLL-like, atypical, and normal lymphocytes) were found in all cases. Thirteen patients (76%) had a majority of CLL-like lymphocytes, whereas the remaining four (24%) had a majority of atypical lymphocytes (Table 1). 

With the exception of one patient who had moderate thrombocytopenia (130 G/L) and anemia (129 g/L), no cytopenia was observed. A monoclonal immunoglobulin was detected by serum electrophoresis in 9/16 patients (56%) with the following isotype on immunofixation: IgG (*n* = 4) or IgM (*n* = 5). β2-microglobulin and lactate dehydrogenase (LDH) were above normal values in 8/10 (80%) and 7/17 (41%) cases, respectively. The direct antiglobulin test was positive in 1 of the 12 patients tested (8%). 

Two patients had a bone marrow biopsy, which showed bone marrow involvement in both cases by an unclassifiable small B-cell lymphoid malignancy.

### 2.2. Immunophenotypic Features 

Among the 18 patients included, 10 (56%) presented with an RMH score of 2, and 8 (44%) with an RMH score of 1. After gating on CD19+ B-cells, 16/18 (89%) cases expressed CD5, 6/18 (33%) expressed CD23, and 4/18 (22%) cases expressed both. There were 3/18 (17%) cases who were CD43 positive, and in most cases (13/18, 72%) CD20 was strongly expressed (Table 1).

### 2.3. Cytogenetic Features 

Clonal chromosomal abnormalities were identified in all patients using conventional karyotype and/or fluorescent in situ hybridization (FISH) targeting 11q22-23, 13q14, and 17p13 loci and chromosome 12 centromeric region. Chromosome 12 trisomy was the most frequent abnormality, having occurred in 13/18 (72%) patients. We observed chromosome 13q deletion and chromosome 17p deletion in 9/18 (50%) and 2/18 (11%) of patients, respectively (Table 1). Two patients presented with a complex karyotype (>3 abnormalities). One patient presented with a t(14;19) [*IGH*/*BCL3*] translocation, associated with chromosome 12 trisomy—an association described in cytologically and immunophenotypically atypical CLL [5].

### 2.4. Gene Mutations

Eight patients were analyzed by the high-throughput DNA sequencing of a large panel of genes recurrently mutated in hematological malignancies (Table S1). Between zero and four mutations per patient were identified (Table 2). Most of these were described in the COSMIC database [6], and all single nucleotide variation was predicted as deleterious and/or disease causing by SIFT and/or Mutation Taster algorithms [7,8]. *TP53* was the most frequently mutated gene (3/8 cases, with 17p deletion in 2 of them). Two patients harbored the same mutation of *MYD88* (p.S219C). *MYD88* mutations areslightly more frequent in marginal zone lymphoma (MZL) than CLL, but one of these two patients also harbored an *SF3B1* mutation, which is far more frequent in CLL (Table S2). The remaining patients harbored mutations found with similar frequencies in both diseases.

### 2.5. IGH Gene Repertoire

Productive clonal *IGH* rearrangements could be obtained for nine cases (Table S3). Based on the percentage of *IGH* variable region (*IGHV*) gene identity to the germline sequences, 7/9 cases could be classified as “significantly mutated” (identity <97%), whereas the two remaining cases were “borderline/minimally mutated” (97–99.9% identity). The majority of cases (6/9) used *IGHV3* subgroup genes while the remaining used *IGHV1* subgroup genes. Among the latter, *IGHV1-2* was identified in two cases—a gene which has been found to be over represented in splenic marginal zone lymphoma (SMZL) [9], although significantly less mutated than in the present study. In contrast, neither of the two predominant genes that contribute to the CLL repertoire [10]—*IGHV1-69* (mostly non-mutated) and *IGHV4-34* (mostly mutated)—were identified in the present cohort.

### 2.6. Clinical Outcome

**Figure 1:**
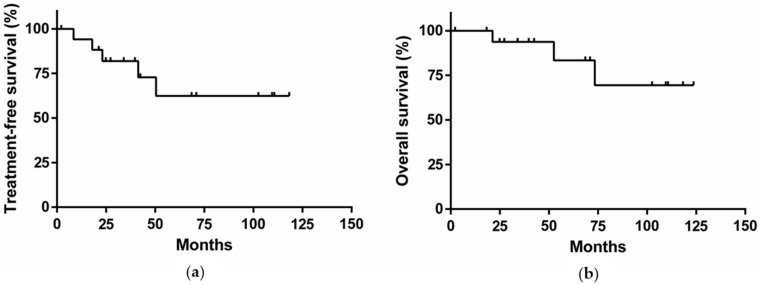
(**a**) Treatment-free survival of the cohort; (**b**) Overall survival of the cohort.

All patients were initially monitored with a wait and watch strategy. After a median follow-up of 48 months, the lymphocyte count doubled in 12/18 patients (67%, median doubling time: 16 months, range: 5–46 months). Eight (44%) patients developed a clinically detectable adenopathy and/or organomegaly (median time to develop clinically detectable adenopathy and/or organomegaly: 22 months; range: 8–48 months). Four patients were treated with chemotherapy or immunochemotherapy, and one underwent splenectomy because of a symptomatic increase of spleen volume associated with anemia (Table 1 and Figure 1a). For these five patients, the median time to first treatment was 23 months. Among them, two patients presented both a chromosome 17p deletion and a *TP53* mutation. Two patients achieved complete remission with a rituximab-chlorambucil regimen, and one achieved partial remission after attenuated R-CHOP (rituximab + cyclophosphamide + doxorubicin + vincristine + prednisone). One patient died a few weeks after initiation of chlorambucil. The splenectomized patient achieved a partial response and a diagnosis of MZL was made on the spleen histology. With a median follow-up of 48 months, the overall survival rate was 83% (Figure 1b). Of note, histological transformation into diffuse large B-cell lymphoma was observed in one patient after two lines of treatment. This patient harbored a chromosome 17p deletion associated with a *TP53* mutation, both known as poor prognosis markers in CLL [11,12].

## 3. Discussion

We report herein a comprehensive description of a cohort of 18 patients with monoclonal lymphocytosis that cannot be accurately classified according to current guidelines [1]. For these patients, the diagnostic procedure was limited to the analysis of circulating lymphocytes or bone marrow because at the time of diagnosis enlarged lymph node, splenomegaly, or hepatomegaly were not detected. This is not a common situation (less than 1/100 CLL diagnoses in our center).

An important question is to what extent these patients can be included within an existing class of patients described by the WHO. Cytological examination of blood smears found in all patients both CLL-like and MZL-like atypical lymphocytes [13]. Immunological features were also shared between CLL (CD5^+^) and MZL (RMH score < 3, CD20^high^, CD43^−^) [3,4,13,14]. Most cases harbored a chromosome 12 trisomy—a cytogenetic feature found in both CLL and MZL but at lower frequencies [11,15,16]. As we found other cytogenetic abnormalities associated with CLL (chromosome 13q deletion and chromosome 17p deletion) [11], but not those associated with MZL (chromosome 3 and 18 trisomies, or chromosome 7q deletion) [15,16,17], the cytogenetic data were more evocative of CLL. High-throughput sequencing did not further resolve the classification of these cases, as most patients had a molecular profile compatible with both CLL and MZL. Note that we did not find *NOTCH1* mutations, which have been associated with chromosome 12 trisomy in CLL [18]. Interestingly, in two of the eight patients for whom a molecular profile was available, we found an *MYD88* p.S219C mutation, which is rarely found in other B-cell neoplasms [12,19,20,21,22,23]. Furthermore, the *IGHV* repertoire, which was mostly mutated, seemed distinct from those reported in CLL [10], MZL [9], or in the recently described clonal B-cell lymphocytosis of marginal zone origin (CBL-MZ) [24]. Finally, the diagnosis of lymphoplasmacytic lymphoma/Waldenström macroglobulinemia seems unlikely, as an *MYD88* mutation was found in only 2/8 patients, and the classical p.L265P mutation was never observed [23].

CBL-MZ was defined as clonal B-cell lymphocytosis with an RMH score < 3 by Xochelli et al. in a population that included patients with absolute lymphocytosis [24]. Most of these patients had negative or weak CD5 staining and had cytogenetic features consistent with MZL, which contrasts with the cohort reported in this paper. These differences might be explained by the exclusion criteria of the present study, in particular the exclusion of lymphocytosis with cytoplasmic projections that were diagnosed as splenic diffuse red pulp small B-cell lymphoma or SMZL according to the WHO classification [1]. However, the only patient in our study with spleen histology was finally diagnosed with an SMZL. An alternative diagnosis is atypical CLL, which is not recognized as an entity in the WHO classification. Atypical CLL refers to clonal absolute lymphocytosis with atypical cytological [25] or immunological features (RMH score < 4) [26,27]. However, the lack of a widely accepted definition has compromised its recognition as a true entity. Interestingly, increased prevalence of chromosome 12 trisomy has been constantly reported in atypical CLL as compared with classical CLL [27], as it was the case in our cohort. Atypical CLL with chromosome 12 trisomy has been associated with t(14;19) [*IGH*/*BCL3*] translocation [5]—a genetic abnormality found in one case of the cohort.

Overall, we report a series of cases displaying intermediate features between CLL and MZL which cannot currently be accurately classified. It is possible that this cohort includes some cases of circulating MZL, expressing CD5 and without classical cytogenetical features diagnosed in early phase, without enlarged lymph node or organomegaly. For example, the patient who underwent a splenectomy received an MZL diagnosis. Only two patients underwent a bone marrow trephine biopsy, which did not allow us to correctly classify the lymphoproliferative disease. However, we cannot exclude the possibility that a bone marrow trephine biopsy would have resulted in a diagnosis for some of these patients. Another limitation of this study is that imaging was not performed for all patients, thus the organomegaly cannot be completely excluded in these cases.

This study highlights limitations of the WHO classification. It provides evidence that refining this classification system is of utmost importance. Larger studies are necessary to precisely know the features of these cases and classify them correctly, especially if a histological diagnosis is not possible. The correct classification of these cases has potential consequences for therapeutic management. Consider the three patients with a *TP53* alteration. The first-line of treatment should be a B-cell receptor inhibitor if considered as CLL [28], whereas there is no first-line indication for these drugs if these pathologies are considered as MZL [2]. Consequently, a histological diagnosis based on bone marrow trephine biopsy must be realized at diagnosis. If treatment is needed, this will allow for the most suitable therapeutic option to be chosen in accordance with the lymphoproliferative disorder involved. However, sometimes this is not sufficient to classify the disease. If lymph node enlargement appears during follow-up and the lymph node is easily accessible, it seems reasonable to complete the diagnostic work-up with the histological analysis of a biopsy. Of note, in our series, the five patients who needed treatment developed a lymph node enlargement or an organomegaly before the treatment began. 

## 4. Materials and Methods

### 4.1. Patients

We selected patients referred to our tertiary care center between 1999 and 2015 who had absolute lymphocytosis (above 5 G/L) due to the presence of monoclonal B cells, but without a clinically detectable enlarged lymph node, splenomegaly, or hepatomegaly. We excluded patients with definitive diagnosis according to the WHO classification [1], such as those with CLL (RMH score ≥ 3) [3], those with MCL (detection of the t(11;14)(q13;q32) translocation) [1], as well as those with cytological, phenotypical, and/or cytogenetical features characteristic of a specific lymphoid neoplasm (e.g., hairy cell leukemia, SMZL, prolymphocytic leukemia). Overall, 18 patients who fulfilled these criteria were identified.

### 4.2. Morphological Analysis

Peripheral blood smears were stained with May–Grünwald–Giemsa and lymphocyte cytology was independently evaluated by two experimented cytologists (MD and LB). Lymphoid cells were classified as CLL-like lymphocytes (i.e., small lymphocytes with a round, clumped nucleus and scanty cytoplasm), atypical lymphocytes (i.e., larger cells with an irregular nucleus and chromatin condensed in small irregular clumps, with more abundant and pale cytoplasm) or cytologically normal lymphocytes.

### 4.3. Flow Cytometry Analysis

Peripheral blood lymphocytes were studied by flow cytometry on a Coulter Epics XL flow cytometer (Beckman Coulter, Brea, CA, USA) or a BD FacsCanto II cytometer (BD Biosciences, San Jose, CA, USA), as previously described [29,30]. The following antigens were detected using monoclonal antibodies: κ, λ, FMC7, CD5, CD19, CD20, CD22, CD23, CD43, and CD79b. Markers were considered positive when expressed by more than 30% of B-cells [3]. The intensity of staining (strong or weak) was defined by the mean fluorescence intensity (MFI, arbitrary units).

### 4.4. Cytogenetic Analysis

Conventional cytogenetic studies were performed on peripheral blood samples after 48–72 h expansion in RPMI 1640 medium supplemented with 12% fetal calf serum, L-glutamine, penicillin, and streptomycin. Cell stimulation was induced using either Phorbol12-myristate 13 acetate (TPA 0.1 µg/mL) or CpG-oligonucleotides added to interleukin 2. Chromosome analyses were carried out on RHG and GBG-banded metaphases as previously described and evaluated according to the International System for Human Cytogenomic Nomenclature recommendations [31,32].

Interphase FISH experiments were performed on chromosome spreads as previously reported using the XL CLL probe kit (XL *DLEU*/*LAMP*/12 cen and XL *ATM*/*TP53)* and the XL *CCND1* break Apart Probe (Metasystems, Altlussheim, Germany) [31].

### 4.5. High-Throughput Sequencing

Libraries were prepared for a custom panel (provided in Table S1) with the Haloplex target enrichment system (Agilent, Santa Clara, CA, USA), according to the supplier’s instructions, and sequenced using a NextSeq 500 (Illumina, San Diego, CA, USA). Raw data from sequencing were analyzed in parallel by two commercial pipelines: Surecall (Agilent, Santa Clara, CA, USA) and Nextgene (SoftGenetics, State College, PA, USA) and manually reviewed. Single-nucleotide polymorphisms with a minor allele frequency above 1% were filtered out.

### 4.6. PCR Amplification and Sequence Analysis of Immunoglobulin Gene Rearrangements 

Amplification of *IGH* gene rearrangements was performed on genomic DNA according to the Biomed2 protocols [33]. Clonal PCR products were purified and directly sequenced on both strands. Sequences were then analyzed using the international ImMunoGeneTics information system (IMGT) database [34] and the IMGT/V-QUEST tool (http://www.imgt.org) [35].

### 4.7. Definitions of Treatment Responses

Treatment response was assessed according to the 2008 guidelines from the International Workshop on Chronic Lymphocytic Leukemia [36]. Treatment-free survival was defined as the interval between diagnosis and first treatment. Overall, survival was defined as the interval between diagnosis and death.

### 4.8. Statistical Analysis

Survival curves were drawn according to the Kaplan–Meier method using GraphPad PRISM 6 software (GraphPad Software, La Jolla, CA, USA). The index date was the date of diagnosis. 

## 5. Conclusions

This work describes a group of patients who expressed intermediate features between recognized WHO entities. Even if this situation is rare, and must occur especially when the evolution seems indolent and explorations are limited, it highlights a limitation of the WHO classification. However, as treatment choice becomes increasingly dependent on classification, a correct diagnosis must be sought in case patients need treatment. Studies of larger cohorts are needed to precisely determine the features of these cases and classify them correctly, especially when a histological diagnosis is not possible.

## Figures and Tables

**Table 1:** Clinico-biological features of the cohort. CLL: Chronic lymphocytic leukemia; UPN: Unique patient number; F: Female; M: Male; ECOG: Eastern Cooperative Oncology Group; RMH: Royal Marsden Hospital; C: Chlorambucil; RC: Rituximab + chlorambucil; aR-CHOP: Attenuated R-CHOP (rituximab + cyclophosphamide + doxorubicin + vincristine + prednisone); Splen.: Splenectomy; CR: Complete response; PR: Partial response

	UPN	1	2	3	4	5	6	7	8	9	10	11	12	13	14	15	16	17	18
**Clinical features**	**Sex**	F	F	M	F	M	M	F	F	F	F	M	M	M	M	F	M	F	F
	**Age** **(years)**	65	74	61	79	70	86	84	92	62	82	66	69	78	81	86	55	81	90
	**ECOG score**	0	0	0	0	0	0	0	0	0	0	0	0	0	0	0	1	0	0
**Blood count**	**Lymphocytes** **(G/L)**	8.39	7.61	8.41	21.32	25.69	11.61	13.23	9.66	5.87	15.63	5.64	9.60	7.45	9.50	11.13	5.30	7.80	14.20
	**Cytology**	CLL-like	CLL-like	At.	CLL-like	**/**	CLL-like	CLL-like	At.	CLL-like	CLL-like	CLL-like	CLL-like	At.	CLL-like	CLL-like	CLL-like	CLL-like	At.
	**Neutrophils** **(G/L)**	4.46	6.30	6.95	7.11	7.01	5.52	4.72	4.61	3.95	6.76	4.98	4.50	5.23	2.38	6.91	9.70	5.45	2.96
	**Hemoglobin** **(g/L)**	130	140	154	130	143	136	141	132	122	142	158	150	152	129	124	146	131	131
	**Platelets** **(G/L)**	257	206	184	386	154	208	246	207	215	240	155	277	247	130	345	468	326	166
**RMH score**	**RMH score**	2	2	1	1	1	1	2	1	2	2	1	2	1	1	2	2	2	2
	**CD5**																		
	**CD23**																		
	**Chain**	λ	κ	λ	κ	λ	λ	λ	κ	λ	κ	κ	λ	κ	κ	λ	κ	κ	κ
	**Fmc7**																		
	**CD22**																		
**Other immunophenotyping markers**	**CD79b**																		
	**CD43**																		
	**CD20**																		
**Cytogenetic features**	**11q deletion**	−	−	−	−	−	−	−	−	−	−	−	−	−	−	−	−	−	−
	**Trisomy 12**	+	+	+	+	−	−	−	+	+	+	−	−	+	+	+	+	+	+
	**13q deletion**	+	+	+	−	+	+	+	+	−	−	+	+	−	−	−	−	−	−
	**17p deletion**	−	−	−	−	+	−	+	−	−	−	−	−	−	−	−	−	−	−
**Clinical outcome**	**Treatment**	No	No	RC	aR-CHOP	Splen.	No	RC	No	No	No	No	No	No	C	No	No	No	No
	**Response**	**/**	**/**	CR	PR	PR	**/**	CR	**/**	**/**	**/**	**/**	**/**	**/**	**/**	**/**	**/**	**/**	**/**
	**Death**	No	No	No	No	No	No	Yes	No	No	No	No	No	No	Yes	No	Yes	No	No
	**Follow-up** **(months)**	118	110	124	18	34	69	73	2	103	111	71	40	42	53	34	21	27	25

CLL-like: Majority of CLL-like lymphocytes on the blood smear; At.: Majority of atypical lymphocytes on the blood smear; blue box: Parameter counting for 1 point in the RMH/modified RMH score (CD5: Positive; CD23: Positive; chain: Weak expression; FMC7: Negative; CD22: Weak expression/negative; CD79b: Negative); green box: Positive (CD43) or strongly expressed (CD20) immunophenotyping parameter; white box: Negative (CD43) or weakly expressed (CD20) immunophenotyping parameter or parameter counting for 0 point in the RMH/modified RMH score; +: Presence of the cytogenetic abnormality; −: Absence of the cytogenetic abnormality.

**Table 2:** Mutational landscape of eight patients. Overview (top) and details (down) of results of the molecular analysis of eight patients. UPN: Unique patient number; VAF: Variant allele frequency.

UPN	*ATM*	*BCOR*	*BIRC3*	*CARD11*	*LYN*	*MYD88*	*SF3B1*	*TP53*	*TRAF2*
**1**									
**2**									
**3**									
**5**									
**6**									
**7**									
**10**									
**14**									
**UPN**	**Gene**	**NM**	**VAF (%)**	**cDNA Change**	**Protein Change**	**Mutation Type**	**COSMIC Database**	**SIFT Prediction**	**Mutation Taster Prediction**
**1**	*BCOR*	NM_001123385.1	49,7	c.3863C>G	p.S1288C	Missense	Not described	Deleterious	Disease causing
**3**	*TRAF2*	NM_021138.3	7,2	c.1210G>A	p.G404R	Missense	Not described	Deleterious	Disease causing
**5**	*TP53*	NM_000546.4	44,5	c.701A>G	p.Y234C	Missense	Described	Deleterious	Disease causing
**5**	*TP53*	NM_000546.4	7,2	c.733G>T	p.G245C	Missense	Described	Deleterious	Disease causing
**6**	*TP53*	NM_000546.4	5,4	c.742C>T	p.R248W	Missense	Described	Deleterious	Disease causing
**6**	*SF3B1*	NM_012433.2	17,3	c.2584G>A	p.E862K	Missense	Described	Deleterious	Disease causing
**6**	*MYD88*	NM_002468.4	19,3	c.656C>G	p.S219C	Missense	Described	Deleterious	Disease causing
**6**	*LYN*	NM_002350.3	5,0	c.602_603del	p.F201Sfs*8	Frameshift	Not described	Frameshift	Frameshift
**7**	*TP53*	NM_000546.4	39,5	c.536A>C	p.H179P	Missense	Described	Deleterious	Disease causing
**7**	*TP53*	NM_000546.4	12,7	c.637C>G	p.R213G	Missense	Described	Deleterious	Disease causing
**10**	*ATM*	NM_000051.3	17,7	c.8560C>T	p.R2854C	Missense	Described	Deleterious	Disease causing
**10**	*BIRC3*	NM_001165.3	6,8	c.1284_1288del	p.E429Gfs*7	Frameshift	Described	Frameshift	Frameshift
**10**	*BIRC3*	NM_001165.3	3,0	c.1639del	p.Q547Nfs*21	Frameshift	Described	Frameshift	Frameshift
**10**	*CARD11*	NM_032415.4	43,2	c.2060C>T	p.A687V	Missense	Described	Tolerated	Disease causing
**14**	*MYD88*	NM_002468.4	27,7	c.656C>G	p.S219C	Missense	Described	Deleterious	Disease causing

## Supplementary Materials

The following are available online at https://www.mdpi.com/2072-6694/11/10/1495/s1, Table S1: Composition of the panel of genes analyzed by high throughput sequencing, Table S2: Prevalence of gene mutations in MZL and CLL, according to published data, Table S3: Sequence analysis of immunoglobulin gene rearrangements for 9 patients.
