# Supplementary material for: Unclassifiable Isolated Monoclonal Lymphocytosis: Comprehensive Description of a Retrospective Cohort
Source: Cancers (Basel). 2019 Oct 4;11(10):1495. doi: 10.3390/cancers11101495 (PMC6826630; doi:10.3390/cancers11101495)
Supplement: Supplementary file 1 [file cancers-11-01495-s001.zip › sup data Degaud et al/Table S1.docx]

| *ABL1* | *BTK* | *CREBBP* | *FLT3* | *KIT* | *NFKBIE* | *PTEN* | *SRSF2* | *TP53* |
| --- | --- | --- | --- | --- | --- | --- | --- | --- |
| *ASXL1* | *CALRET* | *CSF3R* | *FOXO1* | *KMT2A* | *NOTCH1* | *PTPN11* | *STAG1* | *TRAF2* |
| *ASXL2* | *CARD11* | *CSNK1A1* | *GATA2* | *KMT2D* | *NOTCH2* | *RAD21* | *STAG2* | *U2AF1* |
| *ATM* | *CBL* | *CXCR4* | *GNA13* | *KRAS* | *NPM1* | *RUNX1* | *STAT3* | *WT1* |
| *B2M* | *CCND1* | *DNMT3A* | *HRAS* | *LYN* | *NRAS* | *SETBP1* | *STAT6* | *XPO1* |
| *BCL2* | *CCND3* | *DNMT3B* | *ID3* | *MAU2* | *PAX5* | *SF3B1* | *SUZ12* | *ZRSR2* |
| *BCL6* | *CD58* | *EP300* | *IDH1* | *MECOM* | *PDGFRB* | *SH2B3* | *SYK* |  |
| *BCOR* | *CD79A* | *ETNK1* | *IDH2* | *MEF2B* | *PHF6* | *SMC1A* | *TCF3* |  |
| *BCORL1* | *CD79B* | *ETV6* | *IKZF1* | *MPL* | *PIM1* | *SMC3* | *TET2* |  |
| *BIRC3* | *CDKN2A* | *EZH2* | *JAK2* | *MYC* | *PLCG2* | *SMO* | *TNFAIP3* |  |
| *BRAF* | *CEBPA* | *FBXW7* | *KDM6A* | *MYD88* | *PRDM1* | *SOCS1* | *TNFRSF14* |  |

**Table S1:** composition of the panel of genes analyzed by high throughput sequencing
