# Supplementary material for: Unclassifiable Isolated Monoclonal Lymphocytosis: Comprehensive Description of a Retrospective Cohort
Source: Cancers (Basel). 2019 Oct 4;11(10):1495. doi: 10.3390/cancers11101495 (PMC6826630; doi:10.3390/cancers11101495)
Supplement: Supplementary file 1 [file cancers-11-01495-s001.zip › sup data Degaud et al/Table S2.docx]

**Table S2:** prevalence of gene mutations in MZL and CLL, according to published data. MZL: marginal zone lymphoma; CLL: chronic lymphocytic leukemia

| **Gene** | **MZL** | **CLL** |
| --- | --- | --- |
|  | **Mutated cases/all cases (%)** | **Mutated cases/all cases (%)** |
| ***ATM*** | 14/245 (5.7) | 109/966 (11.3) |
| ***BCOR*** | 7/239 (2.9) | 17/966 (1.8) |
| ***BIRC3*** | 21/614 (3.4) | 27/966 (2.8) |
| ***CARD11*** | 28/1066 (2.6) | 10/966 (1.0) |
| ***LYN*** | 1/228 (0.4) | 2/966 (0.2) |
| ***MYD88*** | 137/2060 (6.7) | 32/966 (3.3) |
| ***SF3B1*** | 1/239 (0.4) | 147/966 (15.2) |
| ***TP53*** | 92/1045 (8.8) | 52/966 (5.4) |
| ***TRAF2*** | 4/349 (1.1) | 6/966 (0.6) |

**List of references used to populate the table:**

**Data for CLL:** [1,2]

**Data for MZL:** [3–46]

The mutations frequency in MZL could be underestimated as some publications only research mutations in hotspots, whereas mutations data for CLL come from two whole genome sequencing/whole exome sequencing studies.

1. Landau, D.A.; Tausch, E.; Taylor-Weiner, A.N.; Stewart, C.; Reiter, J.G.; Bahlo, J.; Kluth, S.; Bozic, I.; Lawrence, M.; Böttcher, S.; et al. Mutations driving CLL and their evolution in progression and relapse. *Nature* **2015**, *526*, 525–530.

2. Puente, X.S.; Beà, S.; Valdés-Mas, R.; Villamor, N.; Gutiérrez-Abril, J.; Martín-Subero, J.I.; Munar, M.; Rubio-Pérez, C.; Jares, P.; Aymerich, M.; et al. Non-coding recurrent mutations in chronic lymphocytic leukaemia. *Nature* **2015**, *526*, 519–524.

3. Ansell, S.M.; Secreto, F.J.; Manske, M.; Braggio, E.; Hodge, L.S.; Price-Troska, T.; Ziesmer, S.C.; Chanan-Khan, A.A.; Gertz, M.A.; Dogan, A.; et al. MYD88 Pathway Activation in Lymphoplasmacytic Lymphoma Drives Tumor Cell Growth and Cytokine Expression. *Blood* **2012**, *120*, 2699–2699.

4. Cani, A.K.; Soliman, M.; Hovelson, D.H.; Liu, C.-J.; McDaniel, A.S.; Haller, M.J.; Bratley, J.V.; Rahrig, S.E.; Li, Q.; Briceño, C.A.; et al. Comprehensive genomic profiling of orbital and ocular adnexal lymphomas identifies frequent alterations in MYD88 and chromatin modifiers: new routes to targeted therapies. *Mod. Pathol. Off. J. U. S. Can. Acad. Pathol. Inc* **2016**, *29*, 685–697.

5. Clipson, A.; Wang, M.; de Leval, L.; Ashton-Key, M.; Wotherspoon, A.; Vassiliou, G.; Bolli, N.; Grove, C.; Moody, S.; Escudero-Ibarz, L.; et al. KLF2 mutation is the most frequent somatic change in splenic marginal zone lymphoma and identifies a subset with distinct genotype. *Leukemia* **2015**, *29*, 1177–1185.

6. Gachard, N.; Parrens, M.; Soubeyran, I.; Petit, B.; Marfak, A.; Rizzo, D.; Devesa, M.; Delage-Corre, M.; Coste, V.; Laforêt, M.P.; et al. IGHV gene features and MYD88 L265P mutation separate the three marginal zone lymphoma entities and Waldenström macroglobulinemia/lymphoplasmacytic lymphomas. *Leukemia* **2013**, *27*, 183–189.

7. Ganapathi, K.A.; Jobanputra, V.; Iwamoto, F.; Jain, P.; Chen, J.; Cascione, L.; Nahum, O.; Levy, B.; Xie, Y.; Khattar, P.; et al. The genetic landscape of dural marginal zone lymphomas. *Oncotarget* **2016**, *7*, 43052–43061.

8. Gurth, M.; Bernard, V.; Bernd, H.-W.; Schemme, J.; Thorns, C. Nodal marginal zone lymphoma: mutation status analyses of CD79A, CD79B, and MYD88 reveal no specific recurrent lesions. *Leuk. Lymphoma* **2017**, *58*, 979–981.

9. Hamadeh, F.; MacNamara, S.P.; Aguilera, N.S.; Swerdlow, S.H.; Cook, J.R. MYD88 L265P mutation analysis helps define nodal lymphoplasmacytic lymphoma. *Mod. Pathol. Off. J. U. S. Can. Acad. Pathol. Inc* **2015**, *28*, 564–574.

10. Hockley, S.L.; Else, M.; Morilla, A.; Wotherspoon, A.; Dearden, C.; Catovsky, D.; Gonzalez, D.; Matutes, E. The prognostic impact of clinical and molecular features in hairy cell leukaemia variant and splenic marginal zone lymphoma. *Br. J. Haematol.* **2012**, *158*, 347–354.

11. Insuasti-Beltran, G.; Gale, J.M.; Wilson, C.S.; Foucar, K.; Czuchlewski, D.R. Significance of MYD88 L265P Mutation Status in the Subclassification of Low-Grade B-Cell Lymphoma/Leukemia. *Arch. Pathol. Lab. Med.* **2015**, *139*, 1035–1041.

12. Jiménez, C.; Sebastián, E.; Chillón, M.C.; Giraldo, P.; Mariano Hernández, J.; Escalante, F.; González-López, T.J.; Aguilera, C.; de Coca, A.G.; Murillo, I.; et al. MYD88 L265P is a marker highly characteristic of, but not restricted to, Waldenström’s macroglobulinemia. *Leukemia* **2013**, *27*, 1722–1728.

13. Johansson, P.; Klein-Hitpass, L.; Grabellus, F.; Arnold, G.; Klapper, W.; Pförtner, R.; Dührsen, U.; Eckstein, A.; Dürig, J.; Küppers, R. Recurrent mutations in NF-κB pathway components, KMT2D, and NOTCH1/2 in ocular adnexal MALT-type marginal zone lymphomas. *Oncotarget* **2016**, *7*, 62627–62639.

14. Jung, H.; Yoo, H.Y.; Lee, S.H.; Shin, S.; Kim, S.C.; Lee, S.; Joung, J.-G.; Nam, J.-Y.; Ryu, D.; Yun, J.W.; et al. The mutational landscape of ocular marginal zone lymphoma identifies frequent alterations in TNFAIP3 followed by mutations in TBL1XR1 and CREBBP. *Oncotarget* **2017**, *8*, 17038–17049.

15. Kiel, M.J.; Velusamy, T.; Betz, B.L.; Zhao, L.; Weigelin, H.G.; Chiang, M.Y.; Huebner-Chan, D.R.; Bailey, N.G.; Yang, D.T.; Bhagat, G.; et al. Whole-genome sequencing identifies recurrent somatic NOTCH2 mutations in splenic marginal zone lymphoma. *J. Exp. Med.* **2012**, *209*, 1553–1565.

16. Lenz, G.; Davis, R.E.; Ngo, V.N.; Lam, L.; George, T.C.; Wright, G.W.; Dave, S.S.; Zhao, H.; Xu, W.; Rosenwald, A.; et al. Oncogenic CARD11 mutations in human diffuse large B cell lymphoma. *Science* **2008**, *319*, 1676–1679.

17. Li, Z.-M.; Rinaldi, A.; Cavalli, A.; Mensah, A.A.; Ponzoni, M.; Gascoyne, R.D.; Bhagat, G.; Zucca, E.; Bertoni, F. MYD88 somatic mutations in MALT lymphomas. *Br. J. Haematol.* **2012**, *158*, 662–664.

18. Liu, F.; Karube, K.; Kato, H.; Arita, K.; Yoshida, N.; Yamamoto, K.; Tsuzuki, S.; Kim, W.; Ko, Y.-H.; Seto, M. Mutation analysis of NF-κB signal pathway-related genes in ocular MALT lymphoma. *Int. J. Clin. Exp. Pathol.* **2012**, *5*, 436–441.

19. Martínez, N.; Almaraz, C.; Vaqué, J.P.; Varela, I.; Derdak, S.; Beltran, S.; Mollejo, M.; Campos-Martin, Y.; Agueda, L.; Rinaldi, A.; et al. Whole-exome sequencing in splenic marginal zone lymphoma reveals mutations in genes involved in marginal zone differentiation. *Leukemia* **2014**, *28*, 1334–1340.

20. Moody, S.; Escudero-Ibarz, L.; Wang, M.; Clipson, A.; Ochoa Ruiz, E.; Dunn-Walters, D.; Xue, X.; Zeng, N.; Robson, A.; Chuang, S.-S.; et al. Significant association between TNFAIP3 inactivation and biased IGHV4-34 usage in MALT lymphoma. *J. Pathol.* **2017**.

21. Ngo, V.N.; Young, R.M.; Schmitz, R.; Jhavar, S.; Xiao, W.; Lim, K.-H.; Kohlhammer, H.; Xu, W.; Yang, Y.; Zhao, H.; et al. Oncogenically active MYD88 mutations in human lymphoma. *Nature* **2011**, *470*, 115–119.

22. Novak, U.; Rinaldi, A.; Kwee, I.; Nandula, S.V.; Rancoita, P.M.V.; Compagno, M.; Cerri, M.; Rossi, D.; Murty, V.V.; Zucca, E.; et al. The NF-{kappa}B negative regulator TNFAIP3 (A20) is inactivated by somatic mutations and genomic deletions in marginal zone lymphomas. *Blood* **2009**, *113*, 4918–4921.

23. Ogura, G.; Kikuti, Y.Y.; Kikuchi, T.; Carreras, J.; Sato, T.; Nakamura, N. MYD88 (L265P) Mutation in Malignant Lymphoma Using Formalin-Fixed Paraffin-Embedded Section. *J. Clin. Exp. Hematop. JCEH* **2013**, *53*, 175–177.

24. Ondrejka, S.L.; Lin, J.J.; Warden, D.W.; Durkin, L.; Cook, J.R.; Hsi, E.D. MYD88 L265P somatic mutation: its usefulness in the differential diagnosis of bone marrow involvement by B-cell lymphoproliferative disorders. *Am. J. Clin. Pathol.* **2013**, *140*, 387–394.

25. Parry, M.; Rose-Zerilli, M.J.J.; Gibson, J.; Ennis, S.; Walewska, R.; Forster, J.; Parker, H.; Davis, Z.; Gardiner, A.; Collins, A.; et al. Whole exome sequencing identifies novel recurrently mutated genes in patients with splenic marginal zone lymphoma. *PloS One* **2013**, *8*, e83244.

26. Parry, M.; Rose-Zerilli, M.J.J.; Ljungström, V.; Gibson, J.; Wang, J.; Walewska, R.; Parker, H.; Parker, A.; Davis, Z.; Gardiner, A.; et al. Genetics and Prognostication in Splenic Marginal Zone Lymphoma: Revelations from Deep Sequencing. *Clin. Cancer Res. Off. J. Am. Assoc. Cancer Res.* **2015**, *21*, 4174–4183.

27. Peveling-Oberhag, J.; Wolters, F.; Döring, C.; Walter, D.; Sellmann, L.; Scholtysik, R.; Lucioni, M.; Schubach, M.; Paulli, M.; Biskup, S.; et al. Whole exome sequencing of microdissected splenic marginal zone lymphoma: a study to discover novel tumor-specific mutations. *BMC Cancer* **2015**, *15*, 773.

28. Pham-Ledard, A.; Cappellen, D.; Martinez, F.; Vergier, B.; Beylot-Barry, M.; Merlio, J.-P. MYD88 somatic mutation is a genetic feature of primary cutaneous diffuse large B-cell lymphoma, leg type. *J. Invest. Dermatol.* **2012**, *132*, 2118–2120.

29. Piva, R.; Deaglio, S.; Famà, R.; Buonincontri, R.; Scarfò, I.; Bruscaggin, A.; Mereu, E.; Serra, S.; Spina, V.; Brusa, D.; et al. The Krüppel-like factor 2 transcription factor gene is recurrently mutated in splenic marginal zone lymphoma. *Leukemia* **2015**, *29*, 503–507.

30. Poulain, S.; Roumier, C.; Decambron, A.; Renneville, A.; Herbaux, C.; Bertrand, E.; Tricot, S.; Daudignon, A.; Galiègue-Zouitina, S.; Soenen, V.; et al. MYD88 L265P mutation in Waldenstrom macroglobulinemia. *Blood* **2013**, *121*, 4504–4511.

31. Rossi, D.; Deaglio, S.; Dominguez-Sola, D.; Rasi, S.; Vaisitti, T.; Agostinelli, C.; Spina, V.; Bruscaggin, A.; Monti, S.; Cerri, M.; et al. Alteration of BIRC3 and multiple other NF-κB pathway genes in splenic marginal zone lymphoma. *Blood* **2011**, *118*, 4930–4934.

32. Rossi, D.; Trifonov, V.; Fangazio, M.; Bruscaggin, A.; Rasi, S.; Spina, V.; Monti, S.; Vaisitti, T.; Arruga, F.; Famà, R.; et al. The coding genome of splenic marginal zone lymphoma: activation of NOTCH2 and other pathways regulating marginal zone development. *J. Exp. Med.* **2012**, *209*, 1537–1551.

33. Spina, V.; Khiabanian, H.; Messina, M.; Monti, S.; Cascione, L.; Bruscaggin, A.; Spaccarotella, E.; Holmes, A.B.; Arcaini, L.; Lucioni, M.; et al. The genetics of nodal marginal zone lymphoma. *Blood* **2016**, *128*, 1362–1373.

34. Traverse-Glehen, A.; Bachy, E.; Baseggio, L.; Callet-Bauchu, E.; Gazzo, S.; Verney, A.; Hayette, S.; Jallades, L.; Ffrench, M.; Salles, G.; et al. Immunoarchitectural patterns in splenic marginal zone lymphoma: correlations with chromosomal aberrations, IGHV mutations, and survival. A study of 76 cases. *Histopathology* **2013**, *62*, 876–893.

35. Treon, S.P.; Xu, L.; Yang, G.; Zhou, Y.; Liu, X.; Cao, Y.; Sheehy, P.; Manning, R.J.; Patterson, C.J.; Tripsas, C.; et al. MYD88 L265P Somatic Mutation in Waldenström’s Macroglobulinemia. *N. Engl. J. Med.* **2012**, *367*, 826–833.

36. Trøen, G.; Warsame, A.; Delabie, J. CD79B and MYD88 Mutations in Splenic Marginal Zone Lymphoma. *ISRN Oncol.* **2013**, *2013*, 252318.

37. van den Brand, M.; Rijntjes, J.; Hebeda, K.M.; Menting, L.; Bregitha, C.V.; Stevens, W.B.C.; van der Velden, W.J.F.M.; Tops, B.B.J.; van Krieken, J.H.J.M.; Groenen, P.J.T.A. Recurrent mutations in genes involved in nuclear factor-κB signalling in nodal marginal zone lymphoma-diagnostic and therapeutic implications. *Histopathology* **2017**, *70*, 174–184.

38. van Maldegem, F.; Wormhoudt, T. a. M.; Mulder, M.M.S.; Oud, M.E.C.M.; Schilder-Tol, E.; Musler, A.R.; Aten, J.; Saeed, P.; Kersten, M.J.; Pals, S.T.; et al. Chlamydia psittaci-negative ocular adnexal marginal zone B-cell lymphomas have biased VH4-34 immunoglobulin gene expression and proliferate in a distinct inflammatory environment. *Leukemia* **2012**, *26*, 1647–1653.

39. Varettoni, M.; Arcaini, L.; Zibellini, S.; Boveri, E.; Rattotti, S.; Riboni, R.; Corso, A.; Orlandi, E.; Bonfichi, M.; Gotti, M.; et al. Prevalence and clinical significance of the MYD88 (L265P) somatic mutation in Waldenstrom’s macroglobulinemia and related lymphoid neoplasms. *Blood* **2013**, *121*, 2522–2528.

40. Watkins, A.J.; Huang, Y.; Ye, H.; Chanudet, E.; Johnson, N.; Hamoudi, R.; Liu, H.; Dong, G.; Attygalle, A.; McPhail, E.D.; et al. Splenic marginal zone lymphoma: characterization of 7q deletion and its value in diagnosis. *J. Pathol.* **2010**, *220*, 461–474.

41. Wobser, M.; Maurus, K.; Roth, S.; Appenzeller, S.; Weyandt, G.; Goebeler, M.; Rosenwald, A.; Geissinger, E. Myeloid differentiation primary response 88 mutations in a distinct type of cutaneous marginal-zone lymphoma with a nonclass-switched immunoglobulin M immunophenotype. *Br. J. Dermatol.* **2017**, *177*, 564–566.

42. Xu, L.; Hunter, Z.R.; Yang, G.; Zhou, Y.; Cao, Y.; Liu, X.; Morra, E.; Trojani, A.; Greco, A.; Arcaini, L.; et al. MYD88 L265P in Waldenström macroglobulinemia, immunoglobulin M monoclonal gammopathy, and other B-cell lymphoproliferative disorders using conventional and quantitative allele-specific polymerase chain reaction. *Blood* **2013**, *121*, 2051–2058.

43. Yan, Q.; Huang, Y.; Watkins, A.J.; Kocialkowski, S.; Zeng, N.; Hamoudi, R.A.; Isaacson, P.G.; de Leval, L.; Wotherspoon, A.; Du, M.-Q. BCR and TLR signaling pathways are recurrently targeted by genetic changes in splenic marginal zone lymphomas. *Haematologica* **2012**, *97*, 595–598.

44. Yan, Q.; Wang, M.; Moody, S.; Xue, X.; Huang, Y.; Bi, Y.; Du, M.-Q. Distinct involvement of NF-κB regulators by somatic mutation in ocular adnexal malt lymphoma. *Br. J. Haematol.* **2013**, *160*, 851–854.

45. Zhu, D.; Ikpatt, O.F.; Dubovy, S.R.; Lossos, C.; Natkunam, Y.; Chapman-Fredricks, J.R.; Fan, Y.-S.; Lossos, I.S. Molecular and genomic aberrations in Chlamydophila psittaci negative ocular adnexal marginal zone lymphomas. *Am. J. Hematol.* **2013**, *88*, 730–735.

46. Martinez-Lopez, A.; Curiel-Olmo, S.; Mollejo, M.; Cereceda, L.; Martinez, N.; Montes-Moreno, S.; Almaraz, C.; Revert, J.B.; Piris, M.A. MYD88 (L265P) somatic mutation in marginal zone B-cell lymphoma. *Am. J. Surg. Pathol.* **2015**, *39*, 644–651.
