# Supplementary material for: Unclassifiable Isolated Monoclonal Lymphocytosis: Comprehensive Description of a Retrospective Cohort
Source: Cancers (Basel). 2019 Oct 4;11(10):1495. doi: 10.3390/cancers11101495 (PMC6826630; doi:10.3390/cancers11101495)
Supplement: Supplementary file 1 [file cancers-11-01495-s001.zip › sup data Degaud et al/Table S3.docx]

**Table S3:** sequence analysis of immunoglobulin gene rearrangements for 9 patients. UPN: unique patient number; *IGHV*: immunoglobulin heavy chain (*IGH*) variable region; *IGHD*: *IGH* diversity region; *IGHJ*: *IGH* joining region; HCDR3: heavy chain complementary-determining region 3

| **UPN** | ***IGHV* gene** | ***IGHD* gene** | ***IGHJ* gene** | **% identity** | **Productive rearrangement** | **HCDR3 aminoacid sequence** |
| --- | --- | --- | --- | --- | --- | --- |
| **8** | *IGHV1-2* | *IGHD3-16* | *IGHJ4* | 92,0 | yes | CARGVDLGELSLAPFDSW |
| **3** | *IGHV1-2* | *IGHD6-19* | *IGHJ6* | 91,9 | yes | CARDLHTSGLYGMDVW |
| **7** | *IGHV1-8* | *IGHD2-8* | *IGHJ4* | 99,2 | yes | CARTRSTHGDLDYW |
| **5** | *IGHV3-7* | *IGHD2-21* | *IGHJ4* | 95,7 | yes | CARDGPGGGVVNFDHW |
| **9** | *IGHV3-21* | *IGHD2-21* | *IGHJ5* | 87,3 | yes | CARGGGDIPFDHW |
| **6** | *IGHV3-23* | *IGHD2-15* | *IGHJ6* | 85,9 | yes | CARRDMGLSYMDVW |
| **2** | *IGHV3-53* | *IGHD3-22* | *IGHJ3* | 97,6 | yes | CAREVTNYYYDDTAYPGAFDIW |
| **10** | *IGHV3-72* | *IGHD2-21* | *IGHJ4* | 89,8 | yes | CARVDGHCDGNMCLRVGYW |
| **1** | *IGHV3-74* | *IGHD6-6* | *IGHJ4* | 91,7 | yes | CTRIYISSGYFDYW |
